# Dissemination of *Neisseria gonorrhoeae* with decreased susceptibility to extended-spectrum cephalosporins in Southern China, 2021: a genome-wide surveillance from 20 cities

**DOI:** 10.1186/s12941-023-00587-x

**Published:** 2023-05-17

**Authors:** Yiwen Liao, Qinghui Xie, Xiaoxiao Li, Xiaona Yin, Xingzhong Wu, Mingjing Liu, Yuying Pan, Lihong Zeng, Jianjiang Yang, Zhanqin Feng, Xiaolin Qin, Heping Zheng

**Affiliations:** 1 Dermatology Hospital, Southern Medical University, Lujing Road 2, Yuexiu, Guangzhou, 510091 Guangdong China; 2 Guangzhou Key Laboratory for Sexually Transmitted Diseases Control, Guangzhou, Guangdong People’s Republic of China

**Keywords:** Sexually transmitted infections, *Neisseria gonorrhoeae*, Resistance, Extended-spectrum cephalosporins (ESCs), Genomic epidemiology, Whole-genome sequencing

## Abstract

**Background:**

Antimicrobial resistance (AMR) of untreatable gonococcal infection is an emerging threat, especially in Guangdong, a prosperous province in Southern China.

**Methods:**

*N.gonorrhoeae* was isolated from 20 cities in Guangdong and determined antimicrobial susceptibility. Through whole-genome sequencing (WGS), multilocus sequence typing (MLST), *N.gonorrhoeae* multiantigen sequence typing (NG-MAST), and *N.gonorrhoeae* sequence typing for antimicrobial resistance (NG-STAR) were obtained based on the PubMLST database (https://pubmlst.org/). Phylogenetic analysis was used for dissemination and tracking analysis.

**Results:**

Antimicrobial susceptibility was performed on 347 isolates, and 50 isolates were identified as decreased susceptibility (DS) to cephalosporins. Of which 16.0% (8/50) were ceftriaxone DS, 38.0% (19/50) were cefixime DS, and 46.0% (23/50) were both ceftriaxone and cefixime DS. In all, the dual-resistant rate of the cephalosporin-DS isolates was 96.0% for penicillin and 98.0% for tetracycline-resistant, and 10.0% (5/50) were resistant to azithromycin. All cephalosporin-DS isolates were resistant to ciprofloxacin but sensitive to spectinomycin. The predominant MLSTs were ST7363 (16%, 8/50), ST1903 (14%, 7/50), ST1901 (12%, 6/50), and ST7365 (10%, 5/50). Besides some isolates that failed genotyping (NA), NG-STAR ST1143 (n = 6) and NG-MAST ST17748 (n = 4) were the most prevalent. Twelve isolates with mosaic penA-60.001 allele retained the most elevated cephalosporin MIC (Minimum Inhibitory Concentration). Phylogenetic analysis revealed that epidemic penA-60.001 clones, either domestic or foreign, had spread to nine cities in Guangdong, and 9/12 clones were from the Pearl River Delta region.

**Conclusions:**

*N. gonorrhoeae* with cephalosporins-DS was extensively disseminated in Guangdong, Southern China, requiring strict surveillance.

**Supplementary Information:**

The online version contains supplementary material available at 10.1186/s12941-023-00587-x.

## Introduction

*Neisseria gonorrhoeae* (*N. gonorrhoeae* or gonococcus) is the causative agent of the world's second most common sexually transmitted infectious disease, gonorrhea. Antimicrobial resistance (AMR) of gonococci has become a global public health concern [1, 2]. Extended-spectrum cephalosporins (ESCs) (e.g., ceftriaxone and cefixime) are the only recommended regimen for uncomplicated gonorrhea [3]. However, in recent years, the increasing incidence of cephalosporin treatment failure in patients with gonorrhea has been widely reported [4, 5]. Surveillance data by the China Gonorrhea Resistance Surveillance Project (China-GRSP) showed that the incidence of decreased susceptibility to ceftriaxone increased from 9.7% in 2013 to 12.2% in 2016 [6].

Guangdong, located in southern China, has a population of more than 100 million, of which one-quarter of migrants are from domestic and overseas regions. In 2019, 28,010 cases of gonorrhea were reported in Guangdong, accounting for more than one-fifth (23.7%) of national notifiable cases reported [7]. Our previous study of 4113 isolates collected from nine cities in Guangdong found that the prevalence of ceftriaxone DS increased from 2.05% in 2016 to 16.18% in 2019 and that six strains with penA-60.001 had disseminated among 5/9 cities [8]. To further understand the prevalence of cephalosporin-DS and penA-60.001 clones in the province, isolates were collected from 45 monitoring sites in 20 municipalities. This study highlights the need to reinforce gonococcal surveillance with a focus on ESCs to block the further spread of cephalosporin-DS strains promptly.

## Materials and methods

### Collection of N. gonorrhoeae isolated from 20 cities

Guangdong Province is divided into four regions according to their geographical location: the Pearl River Delta, East, North, and West Guangdong region. Our network, the Guangdong Gonococcal Antibiotic Surveillance Programme (GD-GASP), covers 45 sentinel hospitals in 20 cities. We isolated 174 strains from the Pearl River Delta region, which were Guangzhou (19), Zhongshan (12), Zhuhai (37), Dongguan (11), Shenzhen (22), Jiangmen (20), Foshan (27), Huizhou (12), Zhaoqing (14). In East Guangdong were 62 strains, including Shantou (29), Jieyang (20), and Chaozhou (13). In North Guangdong were 73 strains, that is, Shaoguan (22), Meizhou (18), Qingyuan (27), Heyuan (6), and in West Guangdong were 38 strains: Yunfu (20), Zhanjiang (8), Maoming (8), Yangjiang (2).

### Isolation of N. gonorrhoeae and antimicrobial susceptibility testing

According to the method recommended by the WHO [9], *N. gonorrhoeae* was isolated and incubated in the Thayer-Martin medium (Jinbiao, Zhuhai, China) for 24 h at 36 °C in 5% CO2. Identified the gonococci according to colony morphology, gram staining, oxidase and carbohydrate degradation tests, and systematic identification of biochemical and microbiological tests, as previously described [6, 8]. All isolates were stored at − 80 °C.

The MICs for seven antimicrobials, namely, ceftriaxone, cefixime, penicillin, tetracycline, ciprofloxacin, spectinomycin, and azithromycin, were determined by the agar dilution method [9]. Antimicrobial susceptibilities follow the definition of the Clinical and Laboratory Standards Institute (CLSI) and the European Committee on Antimicrobial Susceptibility Testing (EUCAST) (http://www.eucast.org/clinical_ breakpoints/) [10]. The definition of DS was ceftriaxone MIC ≥ 0.125 mg/L and cefixime MIC ≥ 0.25 mg/L. For quality control, each batch of MIC tests included WHO reference strains: WHO L and ATCC 49226.

### Sequence typing (ST) and AMR determinants

Pure colonies cultured for 18 h were sampled for whole-genome sequencing. DNA was extracted using a customized kit (Promega, Beijing, China) and the library using MGIEasy (lot: 1000006985). The genomes of *N. gonorrhoeae* isolates were sequenced by the BGISEQ DNBSEQ-T7 platform with a 150 bp paired-end strategy (Annoroad, Beijing, China). Reads were de novo assembled by SPAdes (version 3.15.3) and compared to the genome of reference strain FA1090 (Accession GCA_000006845.1). The assembled FASTA files were uploaded to the PubMLST database (https://pubmlst.org/), and then MLST, NG-MAST, and NG-STAR results were obtained. Information regarding mutation or mosaicism of resistance genes (penA, porB, ponA, gyrA, parC, 23SrRNA, etc.) was available in the PubMLST database.

### Phylogenetic analysis

Sequencing reads were mapped to the gonococcal strain FA1090 reference genome using the Burrows-Wheeler alignment (version 0.7.17) “mem” transformation for fast and accurate short-read alignment. SAMtools (version 1.2.1) was used to sort and output calls in variant call format (VCF), and SNP calling was performed by GATK [11]. The final phylogenetic tree (maximum likelihood tree) was constructed by RAxML (version 8.2.12) [12] RaxmlHPC, using the following default parameters: -f a-× 12345 -p 12345 -# 20 -m GTRGAMMA -s -n all -T 20. Tree annotation and optimization were performed by iTOL (https://itol.embl.de/).

Criteria for the strains included in the phylogenetic tree were as follows: the penA-60.001 strains reported in China or globally, of which the SRA data were available on NCBI (National Center for Biotechnology Information), and the original forward and reverse sequencing FASTQ files could be downloaded.

### Statistical analysis

The bar charts and heat maps were performed using using GraphPad Prism version 8.0.1 for Windows (GraphPad Software, San Diego, California, USA). Also, Fisher's exact test analysis was performed by GraphPad Prism 8.0.1. The geometric mean MIC were calculated by Excel software.

## Results

### Susceptibility of ESCs and dual antibiotic resistant

Of 1321 reported cases of gonococci, 347 isolates were monitored for antimicrobiotic susceptibility, with a monitoring rate of 26.27%. Among them, 50 isolates were found to be DSC (decreased susceptibility to cephalosporins), with a DS prevalence of 14.4 (50/347), as shown in Table 1-A. Of which, 16.0% isolates (8/50) were ceftriaxone DS, 38.0% isolates (19/50) were cefixime DS, and 46.0% isolates (23/50) were both ceftriaxone and cefixime DS. Table 1-B shows the proportions that were dual antibiotic resistant in the fifty cephalosporin-DS isolates; 96.0% (48/50) of cephalosporin-DS isolates were simultaneously resistant to penicillin (PEN) and 98.0% (49/50) to tetracycline (TET), ranging from 88.9% to 100.0% in the four regions. All of the cephalosporin-DS isolates were resistant to ciprofloxacin (CIP). Still, all of the strains were sensitive to spectinomycin (SPT), and 10.0% (5/50) of the cephalosporin-DS isolates had azithromycin (AZI) resistance. Among the five isolates with DSC and AZI resistance, two were in the Pearl River Delta region and East Guangdong, respectively, and the rest was in North Guangdong.

**Table 1:** DS to ceftriaxone/cefixime and dual antibiotic resistance of 50 DS isolates

A. Ceftriaxone or/and cefixime DS in different regions							
Region	DSC isolates	Ceftriaxone-DS		Cefixime-DS		Ceftriaxone/Cefixime-DS	
		Isolates	%	Isolates	%	Isolates	%
Pearl River Delta	29	5	17.24	10	34.48	14	48.28
East Guangdong	9	2	22.22	4	44.44	3	33.33
North Guangdong	9	0	0.00	5	55.56	4	44.44
West Guangdong	3	1	33.33	0	0.00	2	66.67
Total	50	8	16.00	19	38.00	23	46.00

B. Dual antibiotic resistance of isolates with DSC in different regions											
Region	DSC isolates	DSC + PEN		DSC + TET		DSC + CIP		DSC + SPT		DSC + AZI	
		Isolates	%	Isolates	%	Isolates	%	Isolates	%	Isolates	%
Pearl river Delta	29	28	96.5	29	100.0	29	100.0	0	0.0	2	6.9
East Guangdong	9	9	100.0	8	88.9	9	100.0	0	0.0	2	22.2
North Guangdong	9	8	88.9	9	100.0	9	100.0	0	0.0	1	11.1
West Guangdong	3	3	100.0	3	100.0	3	100.0	0	0.0	0	0.0
Total	50	48	96.0	49	98.0	50	100.0	0	0.0	5	10.0

*DSC* Decreased susceptibility to cephalosporins (ceftriaxone or cefixime); *CRO* Ceftriaxone; *CFM* Cefixime; *PEN* Penicillin, *TET* Tetracycline, *CIP* Ciprofloxacin, *SPT* Spectinomycin, *AZI* Azithromycin

### Genotyping of MLST, NG-STAR, and NG-MAST

**Fig. 1:**
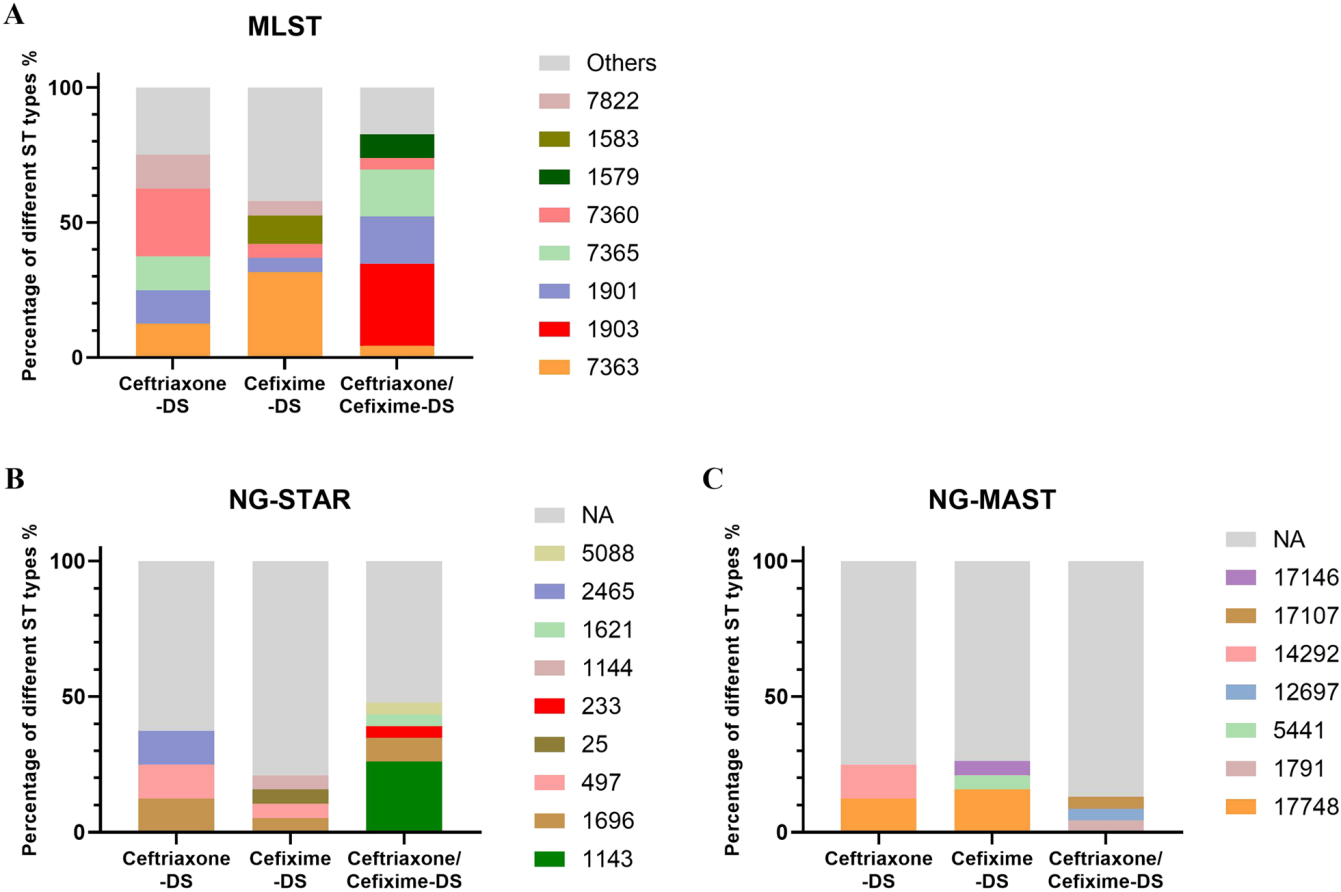
MLST, NG-STAR, and NG-MAST of 50 cephalosporin-DS isolates. Among the 50 ceftriaxone-DS, cefixime-DS, or both ceftriaxone/cefixime-DS isolates, the proportions of different STs based on MLST (**A**), NG-STAR (**B**), and NG-MAST (**C**). NA represents strains that failed typing, and “Others” indicates that the number of isolates in ST typing was less than two

For MLST typing, 50 cephalosporin-DS isolates were assigned to 22 unique STs: ST7363 (16%, 8/50), ST1903 (14%, 7/50), ST1901 (12%, 6/50), ST7365 (10%, 5/50), ST7360 (8%, 4/50), ST1579 (4%, 2/50), ST1583 (4%, 2/50), ST7822 (4%, 2/50), and others STs (28%, 14/50). As shown in Fig. 1A, ST7360 (25.0%, 2/8) was the most common MLST among the eight ceftriaxone-DS isolates. ST7363 (31.6%, 6/19) was popular among the 19 Cefixime-DS isolates. For the 23 Ceftriaxone/Cefixime-DS-DS isolates, ST1903 (30.4%, 7/23) played a dominant role, followed by ST1901 (17.4%, 4/23), ST7365 (17.4%, 4/23), ST1579 (8.7%, 2/23), and other STs (26.1%, 6/23). For NG-STAR typing, 32 isolates failed to type (NA); the rest were NG-STAR ST1143 (n = 6), ST1696 (n = 4), and ST497 (n = 2). For NG-MAST, 40 isolates were NA, and four isolates were NG-MAST ST17748, as shown in Figs. 1B, C. Notably, NG-STAR ST1143 clones were detected only among MLST ST1903 strains, and NG-MAST ST17748 was only detected among MLST ST7363 clones, which can be found in Additional file 1: Table S1.

### Genetic characteristics of resistance

**Fig. 2:**
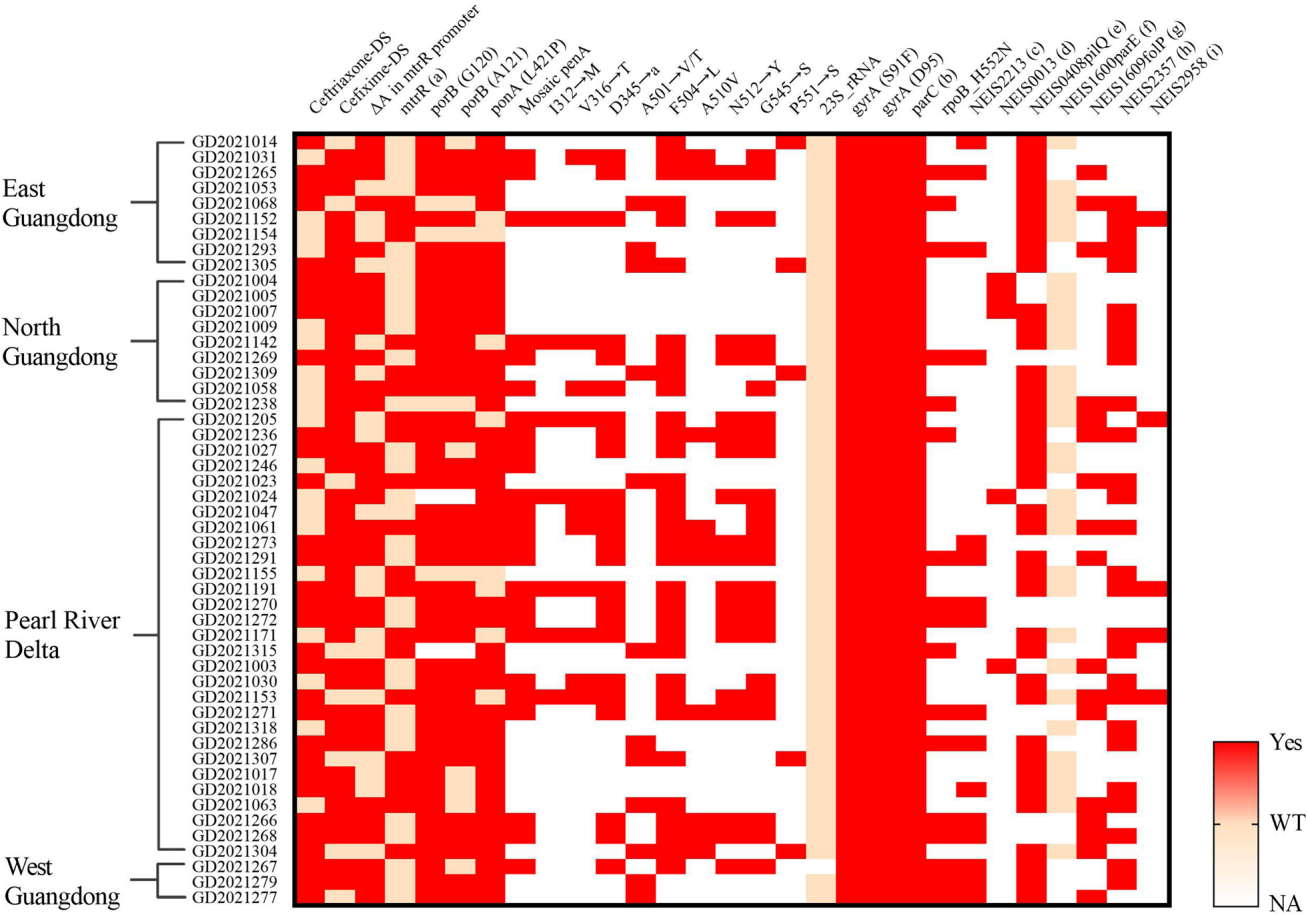
Genetic mutations in the 50 cephalosporin-DS *N. gonorrhoeae* strains from different regions. The vertical axis represents 50 cephalosporin-DS *N. gonorrhoeae* strains and their regional distribution, and the horizontal axis represents the characteristics of each strain, including ceftriaxone DS or cefixime DS and the genetic characteristics. Red represents the presence of cephalosporin DS or mutations (Yes), light pink represents no cephalosporin DS or no mutation, namely Wild Type (WT), and colorless represents NA with unknown information. a: A39T/G45D mutation in mtrR; b: D86/S87 mutation in parC; c: NEIS2213 (zeta_3 toxin variant); d: NEIS0013 (308 bp deletion); e: NEIS0408 (pilQ_S341N/N648S), f: NEIS1600 (parE); g: NEIS1609 (folP_R228S); h: NEIS2357 (TEM-1/TEM-135); h: NEIS2958 (54 bp deletion) mutations

**Fig. 3:**
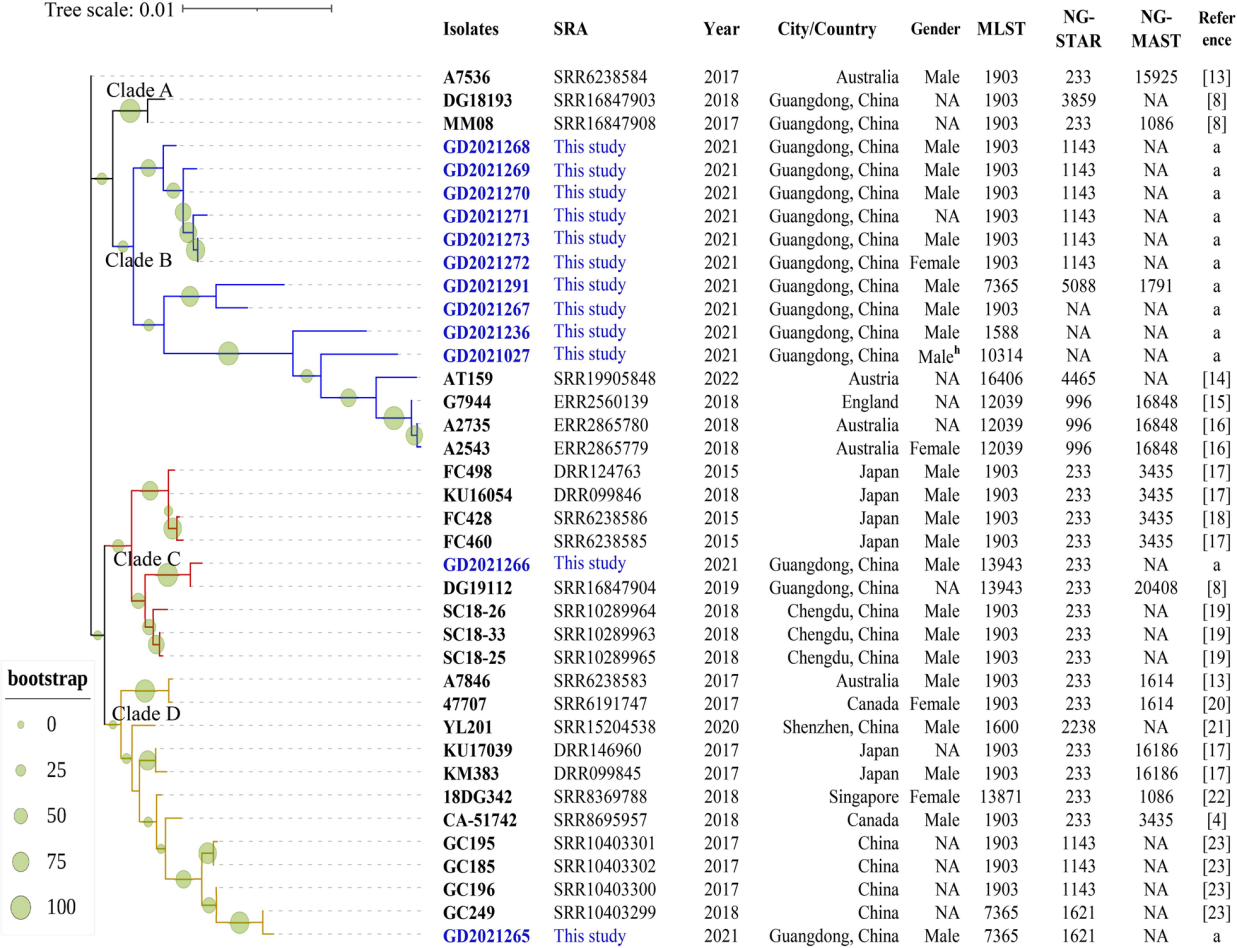
Midpoint-rooted phylogenetic tree of mosaic penA-60.001 *N. gonorrhoeae* clones. Names in blue represent isolates reported in this study; others are isolates previously reported. Year and country show the year and location where isolates were reported. The bootstrap value is labeled with circles of different sizes in green. NA means not available data of gender or NG-STAR or NG-MAST. Reference a: These strains are reported in this study. Male ^h^: the man is reported homosexual

Of the 50 isolates with DS to ceftriaxone or cefixime, 2801 genes were aligned by PubMLST, and mutations of major resistance determinants were found, as shown in Fig. 3 and Additional file 1: Table S1. A total of 64.0% (32/50) of the strains had an adenine deletion (ΔA) in the mtrR promoter. For the ponA gene, 84.0% (42/50) of isolates possessed the L421P mutation, and 16.0% (8/50) were wild-type. For the PorB gene, 88.0% (44/50) possessed the G120D/K mutation, and 76.0% (38/50) had the A121/D/G/N mutation. Other mutants, including gyrA, parC, rpoB_H552N, NEIS2213 (zeta_3 toxin variant), NEIS0013 (308 bp deletion), NEIS0408 (pilQ_S341N/N648S), NEIS1600 (parE), NEIS1609 (folP_R228S), NEIS2357 (TEM-1/TEM-135), and NEIS2958 (54 bp deletion) mutations are shown in Fig. 2.

There were 16 strains with the zeta_3 toxin variant in the NEIS2213 gene, which may be related to ceftriaxone DS. The prevalence of ceftriaxone DS in NEIS2213 mutant strains was 100.0% (16/16), with a geomean MIC of ceftriaxone = 0.3 mg/L, while in strains without the NEIS2213 mutant, the geomean MIC of ceftriaxone = 0.1 mg/L, and the difference was significant (*p* value < 0.05).

### Phylogenetic analysis of penA-60.001 clones

Twelve mosaic penA-60.001 strains were found, and a phylogenetic tree was used to track the evolutionary spread of the clones, as shown in Fig. 3. The phylogenetic tree is roughly divided into four Clades; most of the penA60.001 clones in this study are clustered in Clade B (10/12), and 75% (9/12) belong to the Pearl River Delta region. GD2021236 and GD2021027 are close to the isolates reported in Australia (A2735, A2543) and the United Kingdom (G7944) in 2018. In addition, the isolate (AT159) reported in Austria in 2022 is also close to them. Clade C contains the first reported clone FC428 and related isolates from Japan. The GD2021266 in our study is very close to this subbranch, and was isolated from Zhuhai in the Pearl River Delta of Guangdong. Clade C also contains the DG19112 previously isolated in Guangdong and three isolates (SC18-26, SC18-25, and SC18-33) from Chengdu, Sichuan, China, in 2018. The isolate GD2021265 is distributed in Clade D and adjacent to GC196, GC185, GC195, and GC249 from Changsha, China. They are closely related to the foreign isolates CA-51742 (Canada), 18DG342 (Singapore), KU17039 (Japan), KM383 (Japan), A7846 (Australia), and 47707 (Canada) in 2017 and 2018, as well as YL201 (Shenzhen, China) isolated in 2020, forming an evolutionary Clade D. Detailed information about the twelve strains of penA60.001 is shown in Additional file 2: Table S2 and Additional file 3: Table S3. The mean age of the 12 patients was 31.6 years old, and ten of twelve were male.

## Discussion

AMR surveillance of *N.gonorrhoeae* was highlighted at the 2017 International Forum on Gonorrhea Infection and Drug Resistance [24]. Compared with European [25] and American [26] countries, the AMR of *N.gonorrhoeae* in China is more serious. The present study reveals that in 2021 in Guangdong, there were 50 cephalosporin-DS strains among 347 *N.gonorrhoeae* isolates with a DS prevalence of 14.4% (50/347). What’s more, the prevalence of cephalosporin-DS differs from the region that in the Pearl River Delta, the ceftriaxone-DS and cefixime-DS or both were higher than those in other regions in Guangdong.

The multidrug resistance of gonococci should not be ignored, and the prevalence of penicillin or tetracycline resistance in cephalosporin-DS strains was above 90%. All the cephalosporin-DS isolates were resistant to ciprofloxacin, which may be related to gyrA and parC mutations. All the cephalosporin-DS strains carried the gyrA S91F mutation, and the D86N/S87/S88P mutations in parC were prevalent in the cephalosporin-DS isolates. However, the strains were sensitive to spectinomycin, indicating that spectinomycin can still be used as an alternative in the event of cephalosporin treatment failure. Mutations in PorB (G120K and A121D) slightly reduced susceptibility to penicillin, cefixime, and ceftriaxone, and our data showed that these mutations were prevalent in cephalosporin-DS strains. No C2611T mutation in 23S RNA was found in cephalosporin-DS strains. The prevalence of azithromycin resistance was 10.0%, higher than the WHO recommendation (≥ 5%), which implies that the clinical use of azithromycin may be full of challenges in Guangdong.

It has been reported that the MLST ST1901, ST1903, and ST7363 strains can develop high levels of resistance to cephalosporins [27–29]. In Amsterdam, the Netherlands (2014–2016), ST7363 and ST1901 were principal in the ceftriaxone-DS strains, but ST7827 appeared and dominated in the Netherlands and Europe in 2017–2019 [28]. However, ST7827 was not found in our cephalosporin-DS strains, and our data showed that cephalosporin-DS strains were most associated with ST7363, ST1903, and ST1901. In particular, ST7363 was most related to cefixime-DS strains, similar to the Japanese study, where 61.3% (46/76) of the cefixime-DS isolates were identified as ST7363 [27]. ST7363 showed a global spread and may become the next international cephalosporin-resistant clone after ST1903 [30]. ST7365 was also a common cephalosporin-DS strain, which should cause concern. NG-STAR ST1143 (n = 6) was the prevalent type of cephalosporin-DS strain, and all six strains were penA-60.001 clones. Four cephalosporin-resistant gonococci isolated from China in 2017–2018 were NG-STAR ST1143 [23], which may become an increasingly prevalent cephalosporin-DS strain in China.

Mosaic penA-60.001 clones were the most severe cephalosporin-DS strains in the province. Nine of the twelve penA 60.001 clones were more prevalent in the Pearl River Delta region than in the other regions, possibly due to higher economic development and frequent population mobility. A previous study [6] showed that ceftriaxone-DS strains are more likely to occur in coastal areas because transmission dynamics and sexual networks differ among populations and regions. Studies have shown that the populations of MSM (men who have sex with men) in the economically developed Pearl River Delta region are higher than those in other regions [31], which may be responsible for the increase in cephalosporin-DS strains. The phylogenetic analysis showed that the epidemic penA-60.001 clones from abroad spread to Guangdong. The epidemic strains reported in Sichuan and Hunan in China in recent years have also spread in Guangdong. In addition to the exogenous strains, the native penA-60.001 clones in Guangdong evolved and spread gradually. It was unclear whether these strains acquired mutations through domestic selection pressure or whether they were imported via external inputs. The high prevalence of penA-60.001 clones, especially in the Pearl River Delta region, should be a cause for concern.

## Limitations of the study

The limitations of this study are; first, no further test of isolates with a MIC > 1 mg/l for cephalosporins was carried out to determine high levels of resistance. Second, there may be reporting bias, especially concerning sexual orientation. Since demographic information was collected from participant self-reports and homosexuality is still discriminated against in China, some patients likely concealed their sexual orientation.

## Conclusion

Despite these limitations, our study has meaningfully demonstrated that externally imported and native-evolved cephalosporin-DS strains are extensively disseminated in Guangdong, particularly in the Pearl River Delta region. Our study complements and strengthens AMR's epidemiological and molecular surveillance in Guangdong gonococci.

## Supplementary Information

**Additional file 1: Table S1.**

**Additional file 2: Table S2.** Demographic and clinical information for 12 patients from whom isolated the cephalosporin-resistant penA 60.001 gonococci.

**Additional file 3: Table S3.** Genotypes and resistance determinants of of 12 isolates with cephalosporin-DSC penA 60.001.

## Data Availability

Sequencing raw data of penA-60.001 *Neisseria gonorrhoeae* strains are available at NCBI under BioProject accession number PRJNA957547.

## Abbreviations

DS: Decreased susceptibility
ESCs: Extended-spectrum cephalosporins
AMR: Antimicrobial resistance
WGS: Whole-genome sequencing
MLST: Multilocus sequence typing
NG-MAST: *N. gonorrhoeae* multiantigen sequence typing
NG-STAR: *N.gonorrhoeae* sequence typing for antimicrobial resistance
MIC: Minimum inhibitory concentration
China-GRSP: China Gonorrhea Resistance Surveillance Project
GD-GASP: Guangdong Gonococcal Antibiotic Surveillance Programme
CLSI: Clinical and Laboratory Standards Institute
EUCAST: European Committee on Antimicrobial Susceptibility Testing
DSC: Decreased susceptibility to cephalosporins
CRO: Ceftriaxone
CFM: Cefixime
PEN: Penicillin
TET: Tetracycline
CIP: Ciprofloxacin
SPT: Spectinomycin
AZI: Azithromycin
MSM: Men who have sex with men
