# Supplementary material for: Dissemination of Neisseria gonorrhoeae with decreased susceptibility to extended-spectrum cephalosporins in Southern China, 2021: a genome-wide surveillance from 20 cities
Source: Ann Clin Microbiol Antimicrob. 2023 May 17;22:39. doi: 10.1186/s12941-023-00587-x (PMC10189960; doi:10.1186/s12941-023-00587-x)
Supplement: Supplementary file 2 — Additional file 2: Table S2. Demographic and clinical information for 12 patients from whom isolated the cephalosporin-resistant penA 60.001 gonococci. [file 12941_2023_587_MOESM2_ESM.docx]

| **Supplementary Table 1. Table S2. Demographic and clinical information for 12 patients from whom isolated the cephalosporin-resistant penA 60.001 gonococci.** | | | | | | | | | | | | | | | | | | |
| --- | --- | --- | --- | --- | --- | --- | --- | --- | --- | --- | --- | --- | --- | --- | --- | --- | --- | --- |
|  |  |  |  |  |  |  |  |  |  |  |  |  |  |  |  |  |  |  |
| Sample name | City | Region | Sex | Ethnicity | Sexual orientation | Age | Days infectious with symptoms | Discharge | Sample type | Previous gonorrhea | Antimicrobial use before treatment | PEN (mg/L) | TET (mg/L) | CIP (mg/L) | SPT (mg/L) | CRO (mg/L) | CEF (mg/L) | AZI (mg/L) |
| GD2021236 | dongguan | Pearl River Delta | Male | Han | Heterosexual | 38 | 4 | Yes | Urethral swab | No | No | ≥32 | ≥32 | 8 | 16 | 0.5 | ≥1 | 0.25 |
| GD2021027 | foshan | Pearl River Delta | Male | Han | Homosexual | 19 | 2 | Yes | Urethral swab | No | Yes | ≥32 | 1 | 4 | 16 | 0.5 | ≥1 | 0.25 |
| GD2021273 | guangzhou | Pearl River Delta | Male | NA | Heterosexual | 39 | 2 | Yes | Urethral swab | No | No | 1 | 2 | 16 | 16 | 0.5 | ≥1 | 0.25 |
| GD2021291 | guangzhou | Pearl River Delta | Male | NA | Heterosexual | 32 | NA | Yes | Urethral swab | No | No | 2 | 2 | ≥32 | 16 | 0.5 | ≥1 | 0.25 |
| GD2021270 | huizhou | Pearl River Delta | Male | Han | Heterosexual | 42 | 8 | Yes | Urethral swab | No | No | 4 | 2 | 16 | 16 | 0.5 | ≥1 | 0.5 |
| GD2021272 | huizhou | Pearl River Delta | Female | Han | NA | 39 | 14 | Yes | Cervical swab | No | No | 4 | 2 | 16 | 32 | 0.5 | ≥1 | 0.5 |
| GD2021265 | jieyang | East Guangdong | Male | Han | Heterosexual | 25 | 3 | Yes | Urethral swab | No | No | 4 | 2 | ≥32 | 16 | 0.5 | ≥1 | 2 |
| GD2021267 | maoming | West Guangdong | Male | Han | Heterosexual | 24 | 4 | Yes | Urethral swab | No | No | ≥32 | 2 | ≥32 | 8 | 0.5 | ≥1 | 0.125 |
| GD2021269 | qingyuan | North Guangdong | Male | Han | Heterosexual | 22 | 5 | Yes | Urethral swab | No | No | ≥32 | 1 | 16 | 8 | 0.5 | ≥1 | 0.25 |
| GD2021271 | shenzhen | Pearl River Delta | NA | NA | NA | NA | NA | NA | NA | NA | NA | 4 | 2 | ≥32 | 16 | ≥1 | ≥1 | 0.5 |
| GD2021266 | zhuhai | Pearl River Delta | Male | Han | NA | 21 | 1 | Yes | Urethral swab | No | No | 2 | 2 | 16 | 16 | 0.5 | ≥1 | 0.25 |
| GD2021268 | zhuhai | Pearl River Delta | Male | Han | NA | 47 | 5 | Yes | Urethral swab | No | No | ≥32 | 1 | 8 | 16 | 0.5 | ≥1 | 0.25 |
| PEN, Penicillin; TET, tetracycline; CIP, ciprofloxacin; SPT, spectinomycin; CRO, ceftriaxone; CEF, cefixime; AZI, azithromycin; NA, not available. DS, decreased susceptibility; R, resistance; S susceptibility. | | | | | | | | | | | | | | | | | | |
|  |  |  |  |  |  |  |  |  |  |  |  |  |  |  |  |  |  |  |
