# Supplementary material for: Dissemination of Neisseria gonorrhoeae with decreased susceptibility to extended-spectrum cephalosporins in Southern China, 2021: a genome-wide surveillance from 20 cities
Source: Ann Clin Microbiol Antimicrob. 2023 May 17;22:39. doi: 10.1186/s12941-023-00587-x (PMC10189960; doi:10.1186/s12941-023-00587-x)
Supplement: Supplementary file 3 — Additional file 3: Table S3. Genotypes and resistance determinants of of 12 isolates with cephalosporin-DSC penA 60.001. [file 12941_2023_587_MOESM3_ESM.docx]

| **Table S3. Genotypes and resistance determinants of of 12 isolates with cephalosporin-DSC penA 60.001.** | | | | | | | | | | | | | | | | | | | |
| --- | --- | --- | --- | --- | --- | --- | --- | --- | --- | --- | --- | --- | --- | --- | --- | --- | --- | --- | --- |
|  |  |  |  |  |  |  |  |  |  |  |  |  |  |  |  |  |  |  |  |
| Sample name | NG-STAR | | | | | | | | | | | | | | | NG-MAST | | | MLST |
|  | *penA* | | *mtrR* | | PorB | | PonA | | GyrA | | ParC | | 23srRNA | | NG-STAR | *porB* | *tbpB* | ST |  |
|  | A | R | A | R | A | R | A | R | A | R | A | R | A | R |  |  |  |  |  |
| GD2021236 | 60.001 | Type LX Mosaic | 47 | △A | 12 | G120K, A121G | 1 | L421P | 7 | S91F; D95A | 3 | S87R | 100 | WT | NA | 6931 | 137 | NA | 1588 |
| GD2021027 | 60.001 | Type LX Mosaic | 1 | △A | 4 | G120D | 1 | L421P | 1 | S91F; D95G | 3 | S87R | 100 | WT | NA | 6485 | 75 | NA | 10314 |
| GD2021273 | 60.001 | Type LX Mosaic | 1 | △A | 12 | G120K, A121G | 1 | L421P | 7 | S91F; D95A | 3 | S87R | 100 | WT | 1143 | 3531 | 21 | NA | 1903 |
| GD2021291 | 60.001 | Type LX Mosaic | 1 | △A | 12 | G120K, A121G | 1 | L421P | 21 | S91F; D95A | 3 | S87R | 100 | WT | NA | 543 | 186 | 1791 | 7365 |
| GD2021270 | 60.001 | Type LX Mosaic | 1 | △A | 12 | G120K, A121G | 1 | L421P | 7 | S91F; D95A | 3 | S87R | 100 | WT | 1143 | 3531 | 21 | NA | 1903 |
| GD2021272 | 60.001 | Type LX Mosaic | 1 | △A | 12 | G120K, A121G | 1 | L421P | 7 | S91F; D95A | 3 | S87R | 100 | WT | 1143 | 3531 | 21 | NA | 1903 |
| GD2021265 | 60.001 | Type LX Mosaic | 1 | △A | 12 | G120K, A121G | 13 | L421P | 7 | S91F; D95A | 3 | S87R | 100 | WT | 1621 | NA | NA | NA | 7365 |
| GD2021267 | 60.001 | Type LX Mosaic | 1 | △A | 4 | G120D | 1 | L421P | 3 | S91F; D95A | 3 | S87R | NA | NA | NA | 1973 | 1495 | NA | 1903 |
| GD2021269 | 60.001 | Type LX Mosaic | 1 | △A | 12 | G120K, A121G | 1 | L421P | 7 | S91F; D95A | 3 | S87R | 100 | WT | 1143 | 3531 | 21 | NA | 1903 |
| GD2021271 | 60.001 | Type LX Mosaic | 1 | △A | 12 | G120K, A121G | 1 | L421P | 7 | S91F; D95A | 3 | S87R | 100 | WT | 1143 | 3531 | 21 | NA | 1903 |
| GD2021266 | 60.001 | Type LX Mosaic | 1 | △A | 8 | G120K, A121D | 1 | L421P | 7 | S91F; D95A | 3 | S87R | 100 | WT | 233 | 2035 | 21 | NA | 13943 |
| GD2021268 | 60.001 | Type LX Mosaic | 1 | △A | 12 | G120K, A121G | 1 | L421P | 7 | S91F; D95A | 3 | S87R | 100 | WT | 1143 | NA | 21 | NA | 1903 |
| A, allele type; R: resistance determinant; WT, wild type.  △A, a single nucleotide (A) deletion in mtrR promoter. MLST, multilocus sequence typing; NG-MAST, N. gonorrhoeae multiantigen sequence typing; NG-STAR, N. gonorrhoeae sequence typing for antimicrobial resistance. NA, not available. | | | | | | | | | | | | | | | | | | | |
|  |  |  |  |  |  |  |  |  |  |  |  |  |  |  |  |  |  |  |  |
|  |  |  |  |  |  |  |  |  |  |  |  |  |  |  |  |  |  |  |  |
